# Inter-strain differences of serotonergic inhibitory pain control in inbred mice

**DOI:** 10.1186/1744-8069-6-70

**Published:** 2010-10-26

**Authors:** Nina Wijnvoord, Boris Albuquerque, Annett Häussler, Thekla Myrczek, Laura Popp, Irmgard Tegeder

**Affiliations:** 1 Pharmazentrum frankfurt, ZAFES, Clinical Pharmacology, Goethe-University, Frankfurt, Germany

## Abstract

**Background:**

Descending inhibitory pain control contributes to the endogenous defense against chronic pain and involves noradrenergic and serotonergic systems. The clinical efficacy of antidepressants suggests that serotonin may be particularly relevant for neuropathic pain conditions. Serotonergic signaling is regulated by synthesis, metabolisms, reuptake and receptors.

**Results:**

To address the complexity, we used inbred mouse strains, C57BL/6J, 129 Sv, DBA/2J and Balb/c, which differ in brain serotonin levels. Serotonin analysis after nerve injury revealed inter-strain differences in the adaptation of descending serotonergic fibers. Upregulation of spinal cord and midbrain serotonin was apparent only in 129 Sv mice and was associated with attenuated nerve injury evoked hyperalgesia and allodynia in this strain. The increase of dorsal horn serotonin was blocked by hemisectioning of descending fibers but not by rhizotomy of primary afferents indicating a midbrain source. Para-chlorophenylalanine-mediated serotonin depletion in spinal cord and midbrain intensified pain hypersensitivity in the nerve injury model. In contrast, chronic inflammation of the hindpaw did not evoke equivalent changes in serotonin levels in the spinal cord and midbrain and nociceptive thresholds dropped in a parallel manner in all strains.

**Conclusion:**

The results suggest that chronic nerve injury evoked hypernociception may be contributed by genetic differences of descending serotonergic inhibitory control.

## Background

The development of chronic pain is essentially contributed by loss of inhibitory control of nociceptive signaling mediated by GABAergic or glycinergic interneurons [1-3] and descending inhibitory serotonergic or noradrenergic neurons which originate in the midbrain [4-7]. Pharmacological strengthening of the descending inhibitory pain pathway is thought to contribute to analgesic effects of antidepressants and the opioid agonist tramadol [7,8] suggesting that this inhibitory pathway is clinically relevant particularly for neuropathic pain which in animal models, is associated with profound and long-lasting changes in gene expression [9,10] including those of neuronal tryptophan hydroxylase [11].

In the brain, serotonin is produced by neuronal tryptophan hydroxylase *Tph2 *[12] which also accounts for serotonin synthesis in dorsal root ganglia [11] and is localized within a nociceptive quantitative trait locus [13]. We hypothesized that adaptations of *Tph2*-mediated serotonin synthesis may impact on the efficacy of the descending serotonergic pain control and may contribute to the variability of nociception. However, serotonin plays a multifaceted role in nociceptive excitatory and inhibitory signaling [4,14] and is not only controlled by the rate of its production but also metabolism, reuptake and receptor distribution. Therefore, instead of using a specific deletion model we chose four inbred strains, C57BL/6J, 129 Sv, DBA/2J and Balb/c to assess the in vivo relevance of serotonergic pain control within the context of complex phenotypes. The selection of the strains was based on preliminary screening results which showed differences in brain serotonin levels in these strains, being largely in agreement with data previously found in the striatum of various mouse strains [15] and concentrations in various brain regions in C57Bl/6J mice [16].

Previous studies have revealed that inflammatory pain is strongly controlled by glycinergic inhibition [17] which however does not impact on nerve injury or formalin evoked hypersensitivity [18]. Conversely, nerve injury was associated with GABAergic dysinhibition [1,19]. Hence, depending on the stimulus, inhibitory pain control may involve different in part overlapping or interacting inhibitory circuits. Based on previous studies we hypothesized a strong serotonergic influence on nerve injury evoked hypernociception [20-23]. To address the presumed complexity we assessed nociception in models of chronic inflammatory and neuropathic pain, as well as the formalin assay. In parallel, we analyzed serotonin concentrations assuming that the amount of serotonin at various sites of the brain and spinal cord was best to reflect the availability and turnover of the neurotransmitter.

## Methods

### Animals

Male C57BL/6J, 129 Sv, DBA/2J, and BALB/c mice (Charles River, Sulzfed, Germany), 8-12 weeks old, were housed three to four per cage at constant room temperature (21 ± 1°C) and relative humidity (60%) under a regular light/dark schedule (light on from 7:00 A.M. to 7:00 P.M.) with freely available food and water. Animals were allowed to adapt to laboratory conditions and habituate to the test cages for at least 1 week before starting an experiment with the measurement of baseline nociceptive behavior. Procedures were conducted in conformity with the institutional, national and European laws and policies for animal research and adhered to guidelines set out by the International Association for the Study of Pain (IASP) for the research of pain in conscious animals. They were approved by the local Ethic Committee for Animal Research (Darmstadt, Germany).

### In situ hybridization

Total RNA was extracted from mouse DRG tissue and reversely transcribed with random primers. The fragment was cloned into the pCR4-TOPO plasmid vector (Invitrogen) and sequenced. Riboprobes were obtained by in vitro transcription and labeling with digoxigenin (Dig-labeling kit, Roche).

Fresh frozen DRGs were cut at 14 μm, fixed for 10 min in 4% paraformaldehyde in 0.1 M PBS and acetylated. Sections were prehybridized for 2 h at RT and hybridized at 70°C for 16 h with 200 ng/ml of sense and antisense probes in the prehybridization mix (50% formamide, 5 × SSC, 5 × Denhardt's solution, 500 μg/ml herring sperm DNA, 250 μg/ml yeast tRNA) [11], washed in 0.2% SSC at 60°C and incubated with anti-Dig-AP (1:1000 Roche) in 0.12 M maleic acid buffer with 0.15 M NaCl, pH 7.5 and 1% Blocking Reagent (Roche), washed in TBS, equilibrated in alkaline buffer (0.1 M Tris-HCl, 0.1 M NaCl, 0.05 M MgCl2, pH 9.5, 2 mM levamisole) and developed with NBT/BCIP AP substrate (Roche Diagnostics). Slides were embedded in glycerol/gelatin. Images were obtained using an Eclipse E600 fluorescence microscope equipped with a Kappa DX 20 H camera and Kappa ImageBase software.

### Immunofluorescence

Mice were intracardially perfused with 0.9% saline followed by 4% paraformaldehyde in 0.1 M phosphate buffered saline (PBS, pH 7.4) under terminal isoflurane anesthesia. The L4-5 spinal cord (lumbar enlargement) was removed, postfixed in the same fixative overnight (4°C), embedded with Tissue-Tek^® ^O.C.T. Compound (Science Services, Munich, Germany) and 14 μm thick transversal sections were cut on a cryotome. Sections were incubated in blocking buffer (1% blocking reagent (Roche), 0.05% Triton X-100 in 0.1 M PBS) for 1 h at room temperature and incubated overnight at 4°C with the primary rabbit polyclonal antibody directed against cFos (1:500, Santa Cruz Biotechnology), serotonin (1:500 Abcam) or tyrosine hydroxylase (sheep, 1:500, Abcam). Binding sites were visualized with Cy3 or Alexa-488 conjugated secondary antibodies. Images were obtained with a Zeiss fluorescence microscope. For quantitative analysis of cFos we counted cFos immunoreactive neuronal nuclei in the dorsal horn (superficial and deep layers) of four mice of each strain. Five sections separated by 60-80 μm were counted per mouse and the mean number of cFos IR neurons was used for statistical comparisons. For quantification of serotonin immunoreactivity we used the AutMess Modul of AxioVision (Zeiss). We analyzed the area of 5-HT IR and mean density.

### Serotonin analysis

The brain, spinal cord and dorsal root ganglia (DRGs) were rapidly excised and respective brain regions, L4/5 spinal cord and L4/5 DRGs frozen on dry ice and kept at -80°C until analysis. Serotonin was analyzed by means of a commercial EIA done as recommended by the manufacturer (Labor Diagnostika Nord, Nordhorn, Germany). Serotonin is quantitatively derivatized into N-acylserotonin and then analyzed in a competition EIA microtiter format. Samples of 6 mice of each strain were analyzed for each region.

### Behavioral assessment of nociception

#### Mechanical nociception

We determined the latency of paw withdrawal to a von Frey-like filament using a Dynamic Plantar Aesthesiometer (Ugo Basile, Comerio, Italy). The steel rod was pushed towards the plantar paw. The force increases linearly over 10 sec from 0-5 grams until a strong and immediate withdrawal occurs. In case of no withdrawal up to 5 gram this maximum force is maintained until the paw is withdrawn. The withdrawal latency used for further analysis was the mean of three tests with at least 10 seconds intervals.

#### Thermal nociception

We recorded the withdrawal latency to heat stimulation with a Hot Plate at 52°C, cut-off latency 40 s and a time resolution of 0.1 s (FMI, Föhr Medical Instruments GmbH, Seeheim/Ober-Beerbach, Germany). To measure cold sensitivity and cold allodynia, we recorded the latency for paw withdrawal, licking or jumping after placing the mice onto a Cold Plate that was kept at 10°C (AHP-1200CPHC, Teca, Chicago, IL). The withdrawal latency used for further analysis was the mean of three tests with at least 3 minutes intervals.

#### Chemical nociception

We employed the formalin test to assess chemically evoked acute A-fiber mediated nociception in the first phase and C-fiber sensitization in the second phase of the test [24,25]. We injected 20 μl 5% formalin into the left hindpaw and monitored the licking time for 45 min in 5 min intervals starting directly after injection of the irritant into the paw. The time the mouse spent licking the paw during the first and second phases of the test was used for statistical comparisons.

#### Nerve injury and chronic inflammatory models

Surgery and injections of Complete Freud's Adjuvant (CFA) were carried out under 2% isoflurane anesthesia. For the spared nerve injury (SNI) model of neuropathic pain, two of the three peripheral branches of the sciatic nerve, the common peroneal and the tibial nerves, were ligated with silk (6-0) and distally transected, leaving the sural nerve intact [26,27]. We measured nociceptive behavior (mechanical and cold withdrawal latencies) before and 2-3 times weekly after the nerve injury up to 3 weeks after SNI.

For rhizotomy, we transected the L4 and L5 dorsal roots. The dorsal funiculus was transected at the distal thoracic spinal cord by shallow dorsolateral incision.

For the chronic inflammatory model we injected 20 μl CFA into the plantar side of the left hind paw. Mechanical and heat withdrawal latencies were recorded before and several times after CFA up to 1 week after the injection. Eight mice per group were analyzed in each model.

#### Serotonin depletion in midbrain and spinal cord

4-Chloro-DL-phenylalanine methyl ester hydrochloride (pCPA) was used to deplete serotonin. pCPA (0.4 μg/g/h diluted in Ringer solution) was administered by continuous intrathecal delivery through a spinal catheter with a subcutaneously implanted osmotic pump (Alzet model 2002 for 2 weeks). The control group received vehicle. Eight mice were used per group. In order to enable intrathecal delivery, a polytetrafluoroethylene catheter (PTFE Sub-Lite Wall Tubing 0.05 mm ID × 0.15 mm OD; Braintree Scientific Inc., USA) was stereotactically inserted after distal hemilaminectomy of vertebra L4-L5 under isoflurane anesthesia. The tip of the catheter was slid in rostral direction and positioned above the cervical spinal cord to achieve serotonin depletion in spinal cord and brain areas surrounding the 4^th ^ventricle. The intrathecal catheter was attached to a silicone tube, which was connected to the outlet of the Alzet mini-pump. The Alzet pump was inserted into the subcutaneous space at the left flank. Correct positioning of the catheter tip was checked at the end of the treatment period by microscopic inspection. The efficacy of serotonin depletion was confirmed by serotonin immunostainings of lumbar spinal cord sections.

### Statistics

Data are presented as means ± s.e.m. unless indicated differently. Because of differences in baseline withdrawal thresholds which have been described in detail previously [28,29] we used the relative change of nociceptive thresholds for statistical comparisons. Areas under the curves ("%-change" × "time") were calculated according to the linear trapezoidal rule and subsequently compared by analyses of variances (one-way ANOVA) and post hoc t-tests using a Bonferroni alpha correction. For quantitative analyses of immunostainings we used the AutMess modul of AxioVision software (Zeiss). Serotonin concentrations and counts of c-Fos immunoreactive neurons were also compared with one-way ANOVA and subsequent Bonferroni post hoc analyses, with alpha < 0.05 for all tests.

## Results

### Serotonin concentrations in DRGs and spinal cord

**Figure 1:**
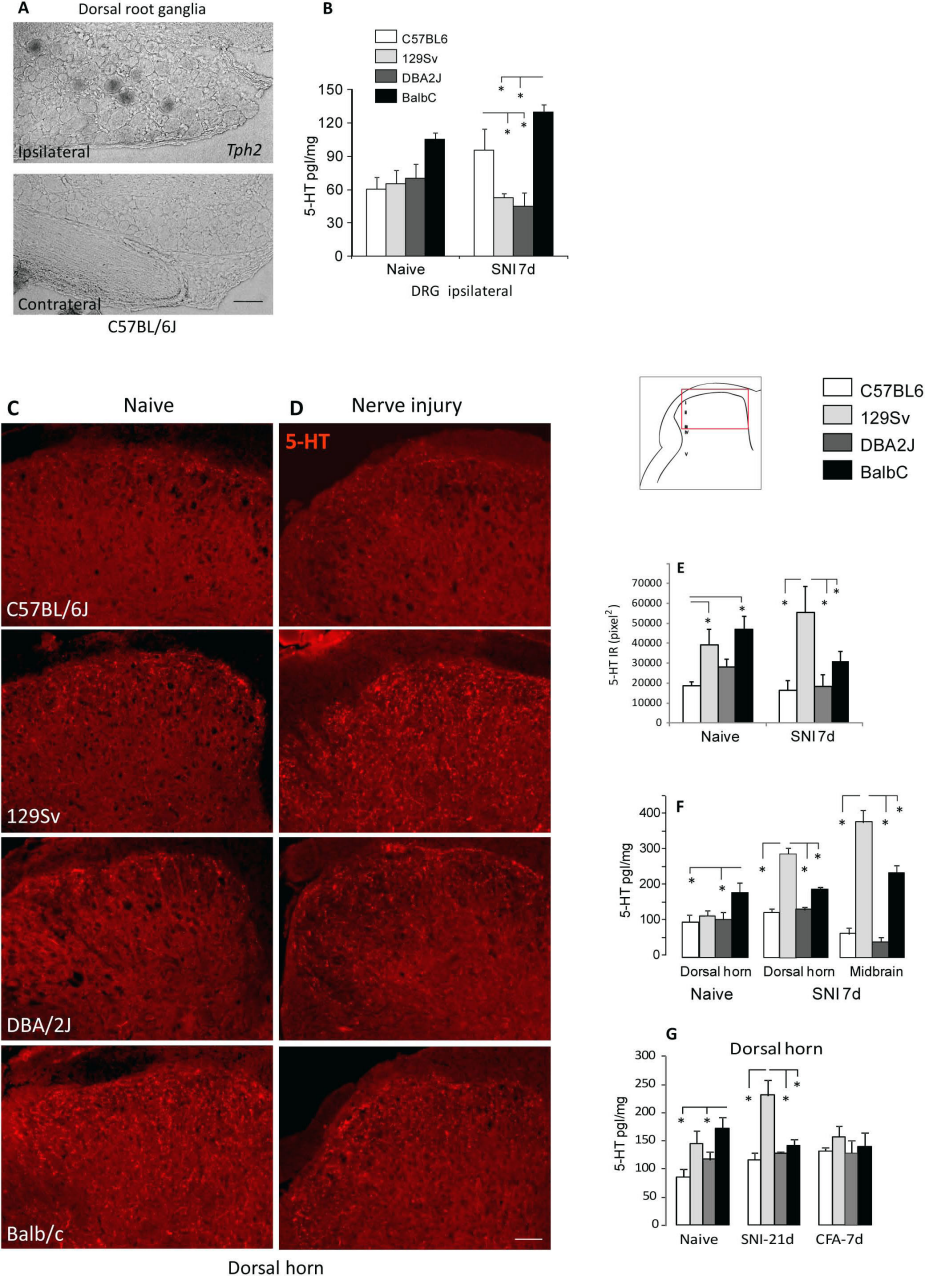
**Regulation of serotonin production in the spinal cord in inbred mouse strains after sciatic nerve injury**. **A **In situ hybridization of *Tph2 *in DRGs on the side ipsilateral and contralateral to a sciatic nerve lesion 7 days after SNI in C57BL/6 mice. Scale bar 50 μm. **B **Serotonin concentrations in DRGs of C57BL/6J, 129 Sv, DBA/2J and Balb/c mice from naïve mice and 7 days after SNI (ipsi lateral L4/5 DRGs). Data are the means ± s.e.m of 4 independent experiments and triplicate analyses. For each experiment pooled tissue from each 3 mice was used. C57BL/6J mice showed a significant increase after SNI. Asterisks indicate statistically significant differences between strains, with P < 0.05. **C-D **Immunofluorescent analysis of serotonin in the L4/5 dorsal horn of the spinal cord of naïve mice and seven days after SNI on the side ipsilateral to the sciatic nerve lesion. Scale bar: 50 μm. **E **Pixel analysis of serotonin immunoreactivity (IR) in the dorsal horn of naïve mice and 7 days after SNI of n = 3 mice per group. Of each mouse 2-3 images were analyzed. **F **Serotonin concentrations in the ipsilateral L4/5 spinal cord dorsal horn and midbrain (region of nucleus raphe magnus) of C57BL/6J, 129 Sv, DBA/2J and Balb/c mice from naïve mice and 7 days after SNI. **G **Serotonin concentrations in the ipsilateral L4/5 spinal cord dorsal horn 21 days after SNI and 7 days after CFA injection into the hindpaw. Concentrations in **F, G **are the means ± sem of 4 independent experiments and triplicate analyses. For each experiment pooled tissue from each 3 mice was used. Asterisks indicate statistically significant differences between strains, with P < 0.05.

To assess expression and adaptation of serotonin in nociceptive pathways we analyzed dorsal root ganglia (DRGs), spinal cord and midbrain in naïve mice and after nerve injury and inflammation. The expression of *Tph2 *in the dorsal root ganglia (DRGs) increased after peripheral nerve injury of the sciatic nerve in the Spared Nerve Injury model (SNI) in C57Bl/6 mice (Figure 1a). We did not detect *Tph1 *in the DRGs. The induction of *Tph2 *was associated with an increase of DRG serotonin concentrations (Figure 1b) in C57BL/6J and Balb/c mice but not in DBA/2J and 129 Sv mice.

At baseline Balb/c mice showed more serotonin immunoreactive fibers in the dorsal horn of the spinal cord as compared to the other strains (Figure 1c, d, e) and higher serotonin concentrations as determined by immunoassays (Figure 1f, g). Sciatic nerve injury evoked a strong increase of dorsal horn serotonin immunoreactivity in 129 Sv mice but not in the other strains (Figure 1d, e). The quantitative immunoassay analysis of serotonin in ipsilateral dorsal horn tissue supported these findings (Figure 1f, g). The increase of serotonin immunoreactivity in 129 Sv mice occurred on both sides, ispilateral and contralateral of the nerve lesion (contra not shown). During chronic inflammatory pain seven days after CFA injection we did not observe significant inter-strain differences of serotonin concentrations neither in dorsal horn nor brainstem (Figure 1g). Baseline differences of serotonin cencentraions in the dorsal horn were apparently lost during inflammation.

**Figure 2:**
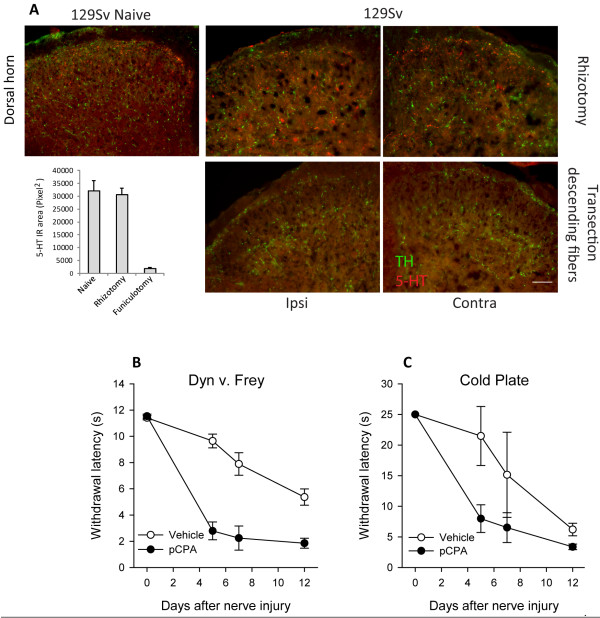
**Serotonin afferents in the spinal cord and effects of serotonin depletion**. **A **Immunofluorescent analysis of serotonin in the L4/5 dorsal horn of the spinal cord seven days after sectioning of the dorsal roots L4 and L5 (Rhizotomy) and 7 days after thoracic transection of descending fibers (dorsolateral corticospinal tract) with tyrosine hydroxylase (TH) counter-staining. Scale bar: 50 μm. The insert shows the quantitative pixel analysis of 5-HT immunoreactive fibers. **B and C **Mechanical and cold nociception in the SNI model following depletion of serotonin in the spinal cord and midbrain. Serotonin depletion was achieved by intrathecal continuous osmotic pump driven infusion of para-chlorophenylalanine (pCPA), an inhibitor of tryptophan hydroxylases through a spinal i.t. catheter. Control animals received vehicle infusions. n = 8 per group. Comparison of AUCs revealed that pCPA-mediated serotonin depletion caused a significant aggravation of nociceptive hypersensitivity after nerve injury.

Serotonin immunoreactivity in 129 Sv mice was not affected by sectioning of the dorsal L4-5 roots (rhizotomy, Figure 2a) but disappeared after unilateral sectioning of the descending dorsal fiber bundles at the distal thoracic level of the spinal cord (Figure 2a). Serotonin immunoreactivity was lost completely on the side ipsilateral to the hemisection, and strongly reduced on the contralateral side suggesting a midbrain source of dorsal horn serotonin.

### Nociception after spinal cord and midbrain depletion of serotonin

We depleted spinal cord and midbrain serotonin by intrathecal delivery of para-chlorophenylalanine (pCPA) and analyzed nociceptive behavior in the SNI model of neuropathic pain (Figure 2b, c). The localization of the catheter tip ensured serotonin depletion in the spinal cord and brain areas surrounding the fourth ventricle. Serotonin immunoreactivity completely disappeared after pCPA treatment (not shown). The observed upregulation of serotonin after nerve injury suggested that the descending serotonergic pain inhibition was relevant mainly for neuropathic pain. We therefore used the SNI model to assess the behavioral effect of serotonin depletion. Mice treated with pCPA showed strongly enhanced mechanical and cold allodynia after SNI as compared to vehicle treatment (p < 0.05 for both stimuli, comparison of AUCs) supporting the idea that serotonin contributed to the endogenous pain control in this model.

### Nerve injury evoked neuropathic pain

**Figure 3:**
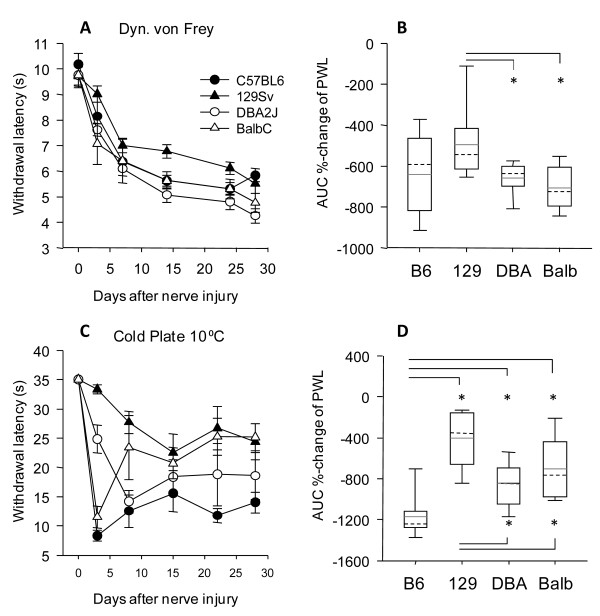
**Nociception in the Spared Nerve Injury model of neuropathic pain in four inbred mouse strains**. **A, C**. Time courses and **B, D **Box Plots of the areas under the curve (time × percentage change) of paw withdrawal latencies in dynamic von Frey and Cold Plate tests in C57BL/6J, 129 Sv, DBA/2J and Balb/c mice before and after sciatic nerve injury in the SNI model. Time course data are means ± s.e.m of n = 8 in each group. Surgery was performed at time "zero" after determination of baseline behavior. Boxes represent the 25^th^-75^th ^percentile, the line represents the median, the dashed line the mean, whisker show the 5^th^-95^th ^percentile. The asterisk indicates statistically significant differences between strains, with P < 0.05.

Based on the observed nerve injury-evoked upregulation of serotonin in 129 Sv mice we hypothesized that this may be associated with a relative protection against neuropathic pain in this strain. We used the Spared Nerve Injury model of neuropathic pain to address this question. During the first week after nerve injury all strains showed a similar drop of the mechanical withdrawal latency (Figure 3a), suggesting that early adaptations were similar in all strains. In the second and third week, withdrawal curves separated (Figure 3a, b). 129 Sv mice were the least sensitive and DBA/2J showed strongest mechanical hypersensitivity. The time curves of cold allodynia revealed immediate inter-strain differences after nerve injury (Figure 3c). Overall, 129 Sv showed the least neuropathic pain-like behavior.

### Formalin evoked nociception and c-Fos induction

**Figure 4:**
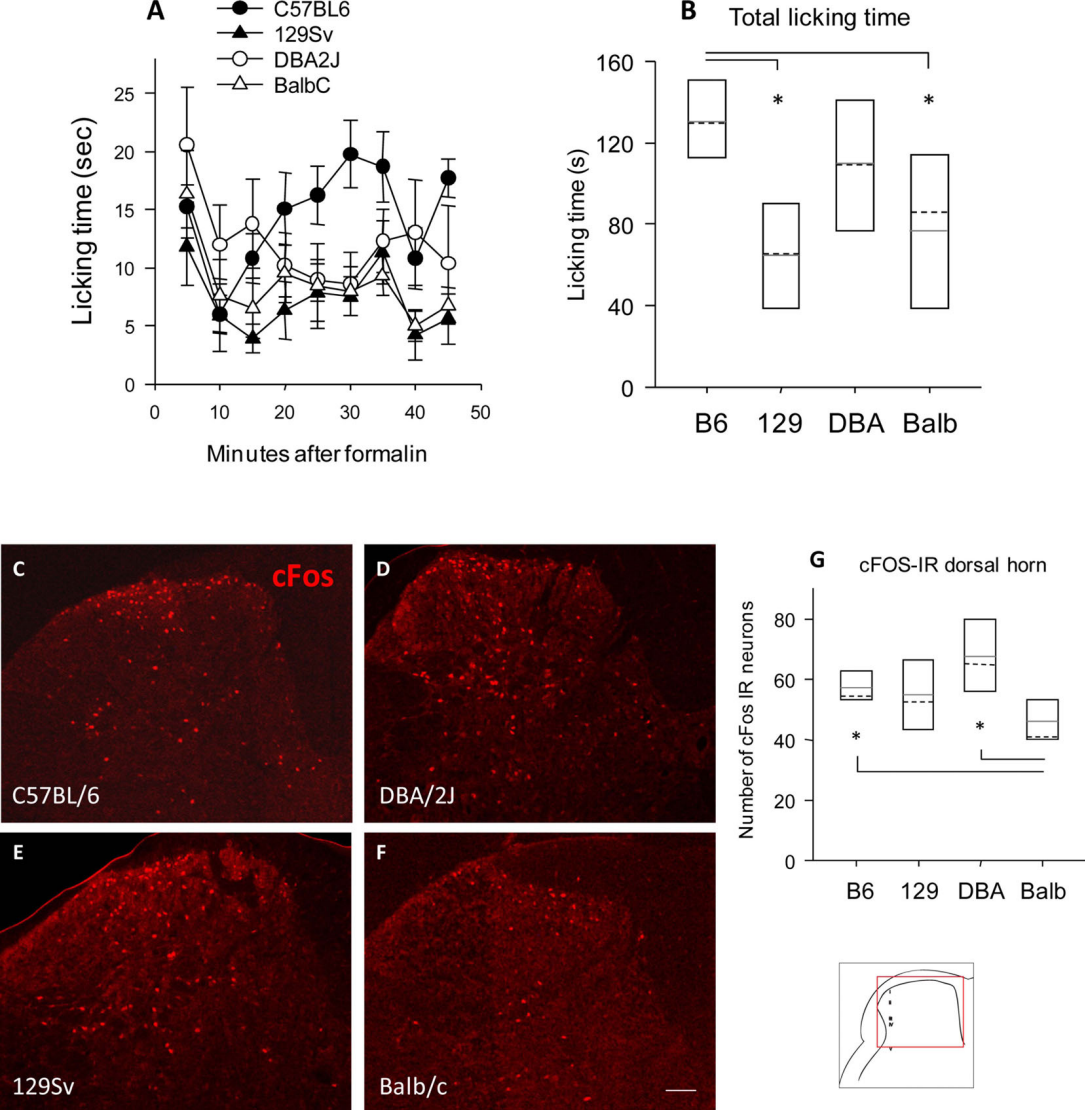
**Nociception in the formalin tests and c-Fos upregulation in the dorsal horn in four inbred mouse strains**. **A**. Chemical nociceptive sensitivity of C57BL/6J, 129 Sv, DBA/2J and Balb/c mice. Data are means ± s.e.m of n = 8 in each group. Nociception was assessed by recording the time course of paw licking behavior after injection of formalin into a hindpaw and **B **comparison of the total licking times. **C-F **Representative results of c-Fos immunoreactivity in the dorsal horn of the spinal cord 2 hours after injection of formalin into the left hindpaw. c-Fos IR neurons were counted of each four C57BL/6J, 129 Sv, DBA/2J and Balb/c mice. Scale bar: 200 μm. **G **Quantitative result of c-Fos IR neurons after formalin injection. The asterisk indicates statistically significant differences between strains, with P < 0.05.

To assess potential inter-strain differences of tonic serotonin-mediated nociceptive inhibition we used the formalin test where the behavioral phenomena occur mainly within the first hour after its injection i.e. before the onset of transcriptional adaptations. We hypothesized that this test may reflect baseline serotonergic differences. C57BL/6J and 129 Sv were the only two lines in which the typical two-phase response was observed, i.e strong licking behavior during the first 5 min after formalin injection, and a second strong licking behavior between 20 and 40 min, separated by a characteristic lack phase between 10 and 15 min. The overall response however, was much stronger in C57BL/6J as compared to 129 Sv and Balb/c mice (Figure 4a, b). DBA/2J showed a late second phase and total licking times were similar to C57BL/6J.

Formalin injection into the hind paw elicits c-Fos upregulation in nociceptive neurons in the dorsal horn of the spinal cord, which has been shown to be genetically determined [30]. The number of c-Fos immunoreactive neurons therefore provides a biochemical indicator for the strength of nociceptive signaling. Representative immunostainings are shown in Figure 4c-f, counts of c-Fos immunoreactive neurons in Figure 4g. In terms of c-Fos activation, Balb/c mice were less sensitive to formalin than C57BL/6J and DBA/2J mice and similar to 129 Sv. Hence, high baseline serotonin concentrations in the spinal cord were associated with a weak formalin-evoked increase of c-Fos immunoreactive neurons.

### Chronic inflammatory hyperalgesia

**Figure 5:**
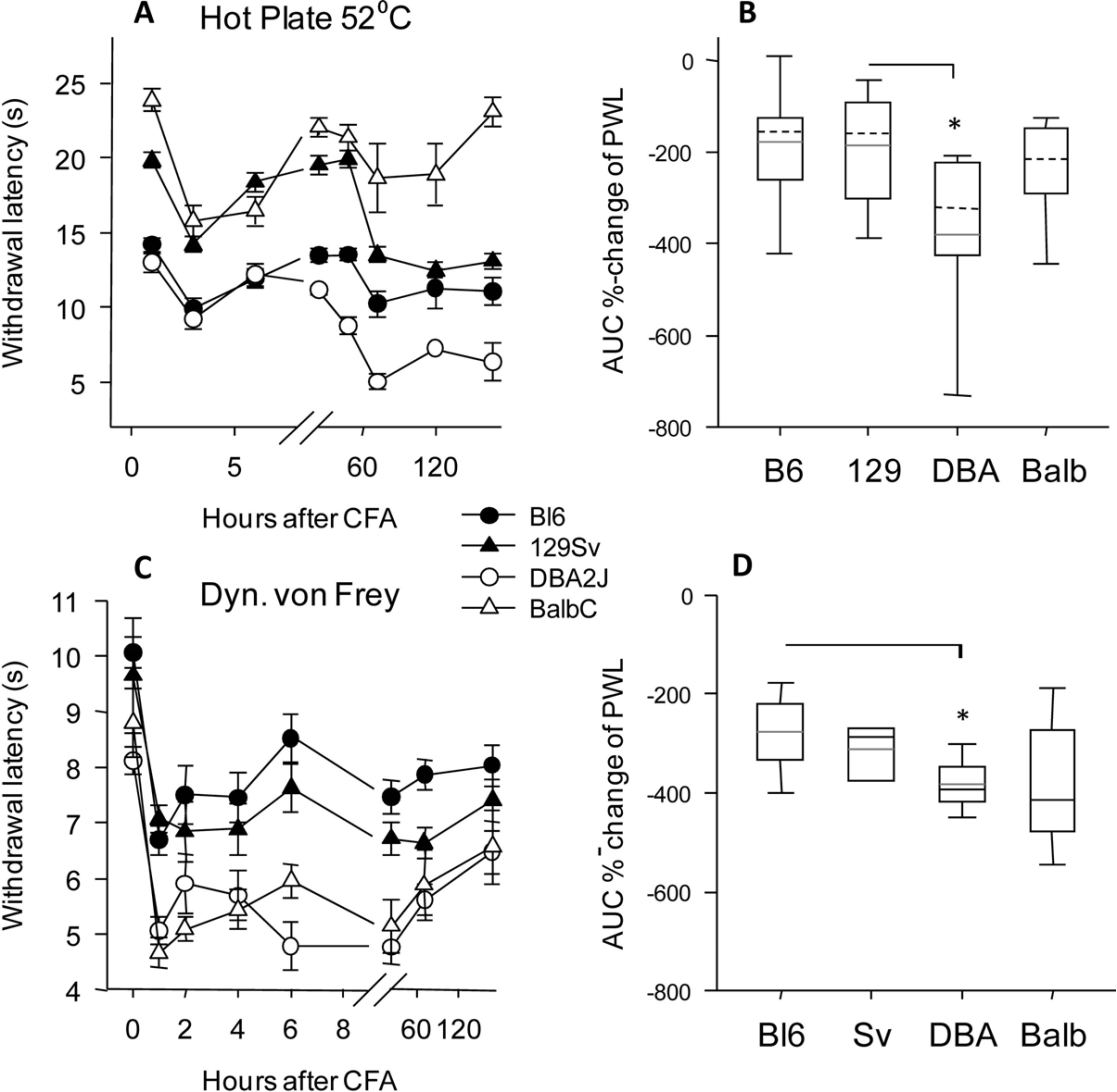
**Inflammatory hyperalgesia in four inbred mouse strains**. **A, C**. Time courses and **B, D **Box plots of the areas under the curve (time × percentage change) of paw withdrawal latencies in Hot Plate and dynamic von Frey tests in C57BL/6J, 129 Sv, DBA/2J and Balb/c mice (data are means ± s.e.m of n = 8 in each group) before and after injection of Complete Freund Adjuvans into one hindpaw. CFA was injected at time "zero" after determination of the baseline. Boxes represent the 25^th^-75^th ^percentile, the line represents the median, the dashed line the mean, whisker show the 5^th^-95^th ^percentile. The asterisk indicates statistically significant differences between strains, with P < 0.05.

Based on the lack of substantial inter-strain differences of spinal cord serotonin concentrations during paw inflammation and loss of baseline differences we hypothesized that behavioral nociceptive adaptations may be more alike among strains than those after nerve injury. As described in detail previously [28,29], heat pain responses at baseline showed remarkable inter-strain differences (Figure 5a). However, after injection of CFA the threshold dropped in a parallel manner in all strains and inter-strain differences only reappeared in the very late phase (Figure 5a). The similarity of the relative change of the threshold was even more obvious in the dynamic von Frey test for mechanical hypersensitivity (Figure 5c). In the first 8 hours after CFA injection, there was no significant difference in the relative decrease of paw withdrawal latencies, neither for thermal nor mechanical stimulation suggesting that the immediate adaptations were similar in all strains tested. Comparison of overall AUCs (Figure 5b, d) revealed stronger inflammatory hypersensitivity of DBA/2J mice as compared to the other strains. Peripheral inflammation itself may follow a genetic program that impacts on the extent of inflammatory hyperalgesia [31].

## Discussion

We observed differences between mouse strains in the adaptations towards nerve injury-evoked nociception, whereas during inflammation thresholds shifted downwards in a parallel manner indicating similar adaptations of inflammatory nociception irrespective of the differences at baseline. For nerve injury-evoked allodynia and formalin-evoked nociception inter-strain differences were not predictable from inter-strain differences of baseline mechanical or thermal sensitivity, but apparently depended on a genetically determined adaptation. Comparisons of serotonin levels in spinal cord and midbrain suggest that the inter-strain differences of chronic neuropathic pain and formalin-evoked nociception were partly contributed by differences in the descending serotonergic inhibitory pain control [32]. 129 Sv mice upregulated serotonin in midbrain and spinal cord after nerve injury and were less sensitive to neuropathic pain compared with the other strains. 129 Sv and particularly Balb/c had high baseline serotonin in the spinal cord and midbrain and relatively weaker nociceptive responses in the second phase of the formalin test suggesting that serotonin may provide some tonic inhibitory control. In peripheral sensory DRGs neurons serotonin is supposed to have mainly excitatory functions [33-37] and may counteract the protective serotonergic effects at spinal cord synapses. Comparisons of serotonin immunoreactivity in the dorsal horn after rhizotomy and after sectioning of descending fiber tracts indicated that the increase of serotonin in the dorsal horn after SNI in 129 Sv mice was mainly mediated by an increase of serotonin at synapses of descending serotonergic neurons in the dorsal horn. Mostly, these descending fibers originate from inhibitory neurons in raphe nuclei of the midbrain and reticular nuclei in the brainstem [38,39]. The observed intensified neuropathic pain like behavior in mice depleted of midbrain and spinal cord serotonin further suggests that the inhibitory pathway is more relevant for the behavioral manifestation of nociception in mice.

Although largely unexplored, it is likely that inbred mouse strains differ in further nociception-relevant differences of neurotransmitter levels or functions that may impact on the behavioral response and efficacy of endogenous inhibitory pain control. Gene expression profiling has revealed some transcriptional differences between strains associated with sensitivity to opioids [40]. Using inbred strains does not allow us to pinpoint the determinant for the observed differences of brain serotonin levels. Transcription of neuronal tryptophan hydroxylase (*Tph2*) was reported not to differ among these mouse strains [41,42]. It should be noted however, that a non-synonymous polymorphism of *Tph2 *(C1473G) was found to determine serotonin production in the mouse brain [42]. The allelic distribution of this SNP among the strains however, is not congruent with brain serotonin levels in the strains. Among the wild types (C57BL/6J and 129 Sv) one strain has low and one high levels. The same holds true for the mutants (DBA/2J and Balb/c). We infer that this SNP, although functional when overexpressed in PC12 cells [42], is not essentially regulating brain serotonin levels in these mice. Supporting this conclusion, knock-in mice of this SNP in a C57BL/6J background did not differ from their wild type littermates neither in terms of serotonin synthesis nor behavior [43]. Nonetheless, other functional differences in Tph2, that remain to be identified, may explain differences of serotonin brain concentrations and the location of *Tph2 *within one of the previously reported nociception-QTLs supports the idea that Tph2-mediated serotonin synthesis is relevant for nociception. In addition, strain specific serotonin metabolism by monoamine oxidases or serotonin N-acetyl transferase may account for differences in serotonin concentrations and behavioral differences, and differences in the serotonin transporter (Slc6A4) may modulate the balance between extracellular and intracellular serotonin [44].

Experiments with mutant mice lacking a specific serotonin receptor or the serotonin transporter have previously provided conflicting results in terms of excitatory and inhibitory functions of serotonin in nociception [6,7,21,23,33-37,45-47]. Although deletion models are non-ambiguous on first view, they are confounded by compensation, background strain and address only one of the complex modifiers of serotonin pathways. For example, monoamine oxidase knockout mice loose 5HT-1A autoreceptors, probably because of the elevated levels of extracellular serotonin [48], surely confounding interpretation of behavioral results with such mice. Hence, the beauty of using inbred mouse strains that differ in the serotonin levels lies in the clear biochemical phenotype on the genetic complexity. Hence, our data suggest that descending serotonergic pain control is genetically determined and contributes to the strain-specific modulation of formalin and nerve injury-evoked nociception.

## Competing interests

The authors declare that they have no competing interests.

## Authors' contributions

NW carried out immunofluorescent studies, BA performed behavioral experiments, AH carried out microsurgery and tissue dissection, TM carried out microscopic slice preparations, LP did behavior experiments, IT conceived of the study, performed surgery, behavior testing, microscopic analyses, analyzed data, and wrote the manuscript. All authors read and approved the final manuscript.

## References

[B1] Knabl J Witschi R Hosl K Reinold H Zeilhofer UB Ahmadi S Brockhaus J Sergejeva M Hess A Brune K Fritschy JM Rudolph U Mohler H Zeilhofer HU Reversal of pathological pain through specific spinal GABAA receptor subtypes Nature 2008 451 7176 330 4 10.1038/nature06493 18202657

[B2] Ahmadi S Lippross S Neuhuber WL Zeilhofer HU PGE(2) selectively blocks inhibitory glycinergic neurotransmission onto rat superficial dorsal horn neurons Nat Neurosci 2002 5 1 34 40 10.1038/nn778 11740501

[B3] Scholz J Broom DC Youn DH Mills CD Kohno T Suter MR Moore KA Decosterd I Coggeshall RE Woolf CJ Blocking caspase activity prevents transsynaptic neuronal apoptosis and the loss of inhibition in lamina II of the dorsal horn after peripheral nerve injury J Neurosci 2005 25 32 7317 23 10.1523/JNEUROSCI.1526-05.2005 16093381 PMC6725303

[B4] Suzuki R Dickenson A Spinal and supraspinal contributions to central sensitization in peripheral neuropathy Neurosignals 2005 14 4 175 81 10.1159/000087656 16215300

[B5] Nichols DS Thorn BE Stimulation-produced analgesia and its cross-tolerance between dorsal and ventral PAG loci Pain 1990 41 3 347 52 10.1016/0304-3959(90)90011-2 2388771

[B6] Peng YB Wu J Willis WD Kenshalo DR GABA(A) and 5-HT(3) receptors are involved in dorsal root reflexes: possible role in periaqueductal gray descending inhibition J Neurophysiol 2001 86 1 49 58 11431487 10.1152/jn.2001.86.1.49

[B7] Zhao ZQ Chiechio S Sun YG Zhang KH Zhao CS Scott M Johnson RL Deneris ES Renner KJ Gereau RWt Chen ZF Mice lacking central serotonergic neurons show enhanced inflammatory pain and an impaired analgesic response to antidepressant drugs J Neurosci 2007 27 22 6045 53 10.1523/JNEUROSCI.1623-07.2007 17537976 PMC6672267

[B8] Berrocoso E De Benito MD Mico JA Role of serotonin 5-HT1A and opioid receptors in the antiallodynic effect of tramadol in the chronic constriction injury model of neuropathic pain in rats Psychopharmacology (Berl) 2007 193 1 97 105 10.1007/s00213-007-0761-8 17393145

[B9] Griffin RS Mills CD Costigan M Woolf CJ Exploiting microarrays to reveal differential gene expression in the nervous system Genome Biol 2003 4 2 105 10.1186/gb-2003-4-2-105 12620110 PMC151293

[B10] Woolf CJ Costigan M Transcriptional and posttranslational plasticity and the generation of inflammatory pain Proc Natl Acad Sci USA 1999 96 14 7723 30 10.1073/pnas.96.14.7723 10393888 PMC33609

[B11] Tegeder I Costigan M Griffin RS Abele A Belfer I Schmidt H Ehnert C Nejim J Marian C Scholz J Wu T Allchorne A Diatchenko L Binshtok AM Goldman D Adolph J Sama S Atlas SJ Carlezon WA Parsegian A Lotsch J Fillingim RB Maixner W Geisslinger G Max MB Woolf CJ GTP cyclohydrolase and tetrahydrobiopterin regulate pain sensitivity and persistence Nat Med 2006 12 11 1269 1277 10.1038/nm1490 17057711

[B12] Walther DJ Peter JU Bashammakh S Hortnagl H Voits M Fink H Bader M Synthesis of serotonin by a second tryptophan hydroxylase isoform Science 2003 299 5603 76 10.1126/science.1078197 12511643

[B13] Wilson SG Chesler EJ Hain H Rankin AJ Schwarz JZ Call SB Murray MR West EE Teuscher C Rodriguez-Zas S Belknap JK Mogil JS Identification of quantitative trait loci for chemical/inflammatory nociception in mice Pain 2002 96 3 385 91 10.1016/S0304-3959(01)00489-4 11973013

[B14] Suzuki R Rygh LJ Dickenson AH Bad news from the brain: descending 5-HT pathways that control spinal pain processing Trends Pharmacol Sci 2004 25 12 613 7 10.1016/j.tips.2004.10.002 15530638

[B15] Mhyre TR Chesler EJ Thiruchelvam M Lungu C Cory-Slechta DA Fry JD Richfield EK Heritability, correlations and in silico mapping of locomotor behavior and neurochemistry in inbred strains of mice Genes Brain Behav 2005 4 4 209 28 15924554 10.1111/j.1601-183X.2004.00102.x

[B16] Devoino LB Al'perina EL Podgornaya EK Polyakov OV Idova GV Il'yuchenok RY Brain content of 5-hydroxytryptamine and 5-hydroxyindoleacetic acid and immune response in aggressive C57bl/6J mice Bull Exp Biol Med 2000 130 10 954 6 10.1023/A:1002853521450 11177291

[B17] Harvey RJ Depner UB Wassle H Ahmadi S Heindl C Reinold H Smart TG Harvey K Schutz B Abo-Salem OM Zimmer A Poisbeau P Welzl H Wolfer DP Betz H Zeilhofer HU Muller U GlyR alpha3: an essential target for spinal PGE2-mediated inflammatory pain sensitization Science 2004 304 5672 884 7 10.1126/science.1094925 15131310

[B18] Hosl K Reinold H Harvey RJ Muller U Narumiya S Zeilhofer HU Spinal prostaglandin E receptors of the EP2 subtype and the glycine receptor alpha3 subunit, which mediate central inflammatory hyperalgesia, do not contribute to pain after peripheral nerve injury or formalin injection Pain 2006 126 1-3 46 53 10.1016/j.pain.2006.06.011 16846696

[B19] Moore KA Kohno T Karchewski LA Scholz J Baba H Woolf CJ Partial peripheral nerve injury promotes a selective loss of GABAergic inhibition in the superficial dorsal horn of the spinal cord J Neurosci 2002 22 15 6724 31 12151551 10.1523/JNEUROSCI.22-15-06724.2002 PMC6758148

[B20] Hains BC Everhart AW Fullwood SD Hulsebosch CE Changes in serotonin, serotonin transporter expression and serotonin denervation supersensitivity: involvement in chronic central pain after spinal hemisection in the rat Exp Neurol 2002 175 2 347 62 10.1006/exnr.2002.7892 12061865

[B21] Alhaider AA Lei SZ Wilcox GL Spinal 5-HT3 receptor-mediated antinociception: possible release of GABA J Neurosci 1991 11 7 1881 8 2066767 10.1523/JNEUROSCI.11-07-01881.1991 PMC6575470

[B22] Suzuki R Morcuende S Webber M Hunt SP Dickenson AH Superficial NK1-expressing neurons control spinal excitability through activation of descending pathways Nat Neurosci 2002 5 12 1319 26 10.1038/nn966 12402039

[B23] Suzuki R Rahman W Rygh LJ Webber M Hunt SP Dickenson AH Spinal-supraspinal serotonergic circuits regulating neuropathic pain and its treatment with gabapentin Pain 2005 117 3 292 303 10.1016/j.pain.2005.06.015 16150546

[B24] Dubuisson D Dennis SG The formalin test: a quantitative study of the analgesic effects of morphine, meperidine, and brain stem stimulation in rats and cats Pain 1977 4 2 161 74 564014 10.1016/0304-3959(77)90130-0

[B25] Tjolsen A Berge OG Hunskaar S Rosland JH Hole K The formalin test: an evaluation of the method Pain 1992 51 1 5 17 10.1016/0304-3959(92)90003-T 1454405

[B26] Decosterd I Woolf CJ Spared nerve injury: an animal model of persistent peripheral neuropathic pain Pain 2000 87 2 149 58 10.1016/S0304-3959(00)00276-1 10924808

[B27] Bourquin AF Suveges M Pertin M Gilliard N Sardy S Davison AC Spahn DR Decosterd I Assessment and analysis of mechanical allodynia-like behavior induced by spared nerve injury (SNI) in the mouse Pain 2006 122 1-2 14 e1-14 10.1016/j.pain.2005.10.036 16542774

[B28] Mogil JS Wilson SG Bon K Lee SE Chung K Raber P Pieper JO Hain HS Belknap JK Hubert L Elmer GI Chung JM Devor M Heritability of nociception II. 'Types' of nociception revealed by genetic correlation analysis Pain 1999 80 1-2 83 93 10.1016/S0304-3959(98)00196-1 10204720

[B29] Mogil JS Wilson SG Bon K Lee SE Chung K Raber P Pieper JO Hain HS Belknap JK Hubert L Elmer GI Chung JM Devor M Heritability of nociception I: responses of 11 inbred mouse strains on 12 measures of nociception Pain 1999 80 1-2 67 82 10.1016/S0304-3959(98)00197-3 10204719

[B30] Bon K Wilson SG Mogil JS Roberts WJ Genetic evidence for the correlation of deep dorsal horn Fos protein immunoreactivity with tonic formalin pain behavior J Pain 2002 3 3 181 9 10.1054/jpai.2002.123710 14622771

[B31] Hoover-Plow JL Gong Y Shchurin A Busuttil SJ Schneeman TA Hart E Strain and model dependent differences in inflammatory cell recruitment in mice Inflamm Res 2008 57 10 457 63 10.1007/s00011-008-7062-5 18827970 PMC3031869

[B32] Lopez-Garcia JA King AE Pre- and post-synaptic actions of 5-hydroxytryptamine in the rat lumbar dorsal horn in vitro: implications for somatosensory transmission Eur J Neurosci 1996 8 10 2188 97 10.1111/j.1460-9568.1996.tb00740.x 8921310

[B33] Linhart O Obreja O Kress M The inflammatory mediators serotonin, prostaglandin E2 and bradykinin evoke calcium influx in rat sensory neurons Neuroscience 2003 118 1 69 74 10.1016/S0306-4522(02)00960-0 12676138

[B34] Okamoto K Imbe H Morikawa Y Itoh M Sekimoto M Nemoto K Senba E 5-HT2A receptor subtype in the peripheral branch of sensory fibers is involved in the potentiation of inflammatory pain in rats Pain 2002 99 1-2 133 43 10.1016/S0304-3959(02)00070-2 12237191

[B35] Mamet J Baron A Lazdunski M Voilley N Proinflammatory mediators, stimulators of sensory neuron excitability via the expression of acid-sensing ion channels J Neurosci 2002 22 24 10662 70 12486159 10.1523/JNEUROSCI.22-24-10662.2002 PMC6758460

[B36] Tokunaga A Saika M Senba E 5-HT2A receptor subtype is involved in the thermal hyperalgesic mechanism of serotonin in the periphery Pain 1998 76 3 349 55 10.1016/S0304-3959(98)00066-9 9718253

[B37] Gold MS Reichling DB Shuster MJ Levine JD Hyperalgesic agents increase a tetrodotoxin-resistant Na+ current in nociceptors Proc Natl Acad Sci USA 1996 93 3 1108 12 10.1073/pnas.93.3.1108 8577723 PMC40039

[B38] Braz JM Basbaum AI Genetically expressed transneuronal tracer reveals direct and indirect serotonergic descending control circuits J Comp Neurol 2008 507 6 1990 2003 10.1002/cne.21665 18273889 PMC4947410

[B39] Braz JM Enquist LW Basbaum AI Inputs to serotonergic neurons revealed by conditional viral transneuronal tracing J Comp Neurol 2009 514 2 145 60 10.1002/cne.22003 19274668 PMC2696271

[B40] Tapocik JD Letwin N Mayo CL Frank B Luu T Achinike O House C Williams R Elmer GI Lee NH Identification of candidate genes and gene networks specifically associated with analgesic tolerance to morphine J Neurosci 2009 29 16 5295 307 10.1523/JNEUROSCI.4020-08.2009 19386926 PMC2933065

[B41] Cervo L Canetta A Calcagno E Burbassi S Sacchetti G Caccia S Fracasso C Albani D Forloni G Invernizzi RW Genotype-dependent activity of tryptophan hydroxylase-2 determines the response to citalopram in a mouse model of depression J Neurosci 2005 25 36 8165 72 10.1523/JNEUROSCI.1816-05.2005 16148224 PMC6725548

[B42] Zhang X Beaulieu JM Sotnikova TD Gainetdinov RR Caron MG Tryptophan hydroxylase-2 controls brain serotonin synthesis Science 2004 305 5681 217 10.1126/science.1097540 15247473

[B43] Tenner K Qadri F Bert B Voigt JP Bader M The mTPH2 C1473G single nucleotide polymorphism is not responsible for behavioural differences between mouse strains Neurosci Lett 2008 431 1 21 5 10.1016/j.neulet.2007.11.012 18082956

[B44] Carneiro AM Airey DC Thompson B Zhu CB Lu L Chesler EJ Erikson KM Blakely RD Functional coding variation in recombinant inbred mouse lines reveals multiple serotonin transporter-associated phenotypes Proc Natl Acad Sci USA 2009 106 6 2047 52 10.1073/pnas.0809449106 19179283 PMC2632716

[B45] Berrocoso E Rojas-Corrales MO Mico JA Differential role of 5-HT1A and 5-HT1B receptors on the antinociceptive and antidepressant effect of tramadol in mice Psychopharmacology (Berl) 2006 188 1 111 8 10.1007/s00213-006-0464-6 16832657

[B46] Suzuki R Rahman W Hunt SP Dickenson AH Descending facilitatory control of mechanically evoked responses is enhanced in deep dorsal horn neurones following peripheral nerve injury Brain Res 2004 1019 1-2 68 76 10.1016/j.brainres.2004.05.108 15306240

[B47] Xiao DQ Zhu JX Tang JS Jia H 5-hydroxytryptamine 1A (5-HT1A) but not 5-HT3 receptor is involved in mediating the nucleus submedius 5-HT-evoked antinociception in the rat Brain Res 2005 1046 1-2 38 44 10.1016/j.brainres.2005.03.028 15869749

[B48] Lanoir J Hilaire G Seif I Reduced density of functional 5-HT1A receptors in the brain, medulla and spinal cord of monoamine oxidase-A knockout mouse neonates J Comp Neurol 2006 495 5 607 23 10.1002/cne.20916 16498683

